# Neurological and neuropsychiatric comorbidities occurring in fatty liver diseases

**DOI:** 10.1192/j.eurpsy.2023.1945

**Published:** 2023-07-19

**Authors:** I. M. Balmus, M. -.-A. Robea, R. Lefter, A. Ciobica, L. Huiban, C. Stanciu, A. Trifan

**Affiliations:** 1 Department of Gastroenterology - Faculty of Medicine, Gr. T. Popa University of Medicine and Pharmacy; 2 Center of Biomedical Research, Romanian Academy, Iasi Branch; 3 Department of Biology, Faculty of Biology, Alexandru Ioan Cuza University of Iasi, Iasi, Romania

## Abstract

**Introduction:**

The most common liver diseases associated with the fat accumulation in the hepatic tissue are metabolic associated fatty liver disease (MAFLD), non-alcoholic fatty liver disease (NAFLD), and non-alcoholic steatohepatitis (NASH). Many studies previously reported several key mechanisms tying hepatic injury to extrahepatic manifestations. In this way, the co-occurrence of cognitive decline, mood, and affective changes could suggest the existence of a strong neurological component predisposing to neuropsychiatric comorbidities.

**Objectives:**

In this study, we aimed to describe the neurological and neuropsychiatric comorbidities of MALFD, NAFLD, and NASH.

**Methods:**

The main scientific databases were screened for English-written studies using the following key words: ”cognitive decline”, ”neuronal loss”, ”affective disorders”, ”anxiety”, ”depression”, ”MAFLD”, ”NAFLD”, ”NASH”. Exclusion criteria: (1) studies not focussing on fatty liver diseases; (2) not describing comorbid conditions; (3) not providing correlative analysis of disease co-occurrence or mechanistic associations.

**Results:**

Hepatic encephalopathy (HE) is the main NAFLD/NASH extrahepatic manifestation commonly characterized by impaired cognition, rapid mood swings, depressive and anxious behaviours, and defective sleep. It is currently reported that more than 70% of the cirrhotic patients develop HE. Cognitive impairments and brain tissue reduction were found in NAFLD patients, while MAFLD patients’ cognitive dysfunctions (mild cognitive impairment and hippocampal-dependent memory impairment) were not associated with the presence of metabolic syndrome. Similarly Alzheimer’s disease (AD) was not described as comorbid in MAFLD. By contrast, since NAFLD and NASH are often characterized by insulin resistance and dyslipidaemia – significant triggers of dementia. By far the most prevalent neuropsychiatric comorbidity in NAFLD and NASH is the major depressive syndrome, diagnosed in almost 30% of the cases. Also, a correlation between the anxiety manifestation and the progression from NAFLD to NASH was described. In this context, as a response to the vast evidence that connect liver dysfunction to cognitive impairments, the liver-brain axis function was hypothesized.

**Conclusions:**

MAFLD, NAFLD, and NASH are frequently associated with cognitive decline. The main NASH neurological comorbidity is hepatic encephalopathy, but it could also be seen in NAFLD. While Alzheimer’s disease occurs in NAFLD and NASH, more studies are needed to explain the severity-dependent association. Depression and anxiety were also reported in NAFLD and NASH.

Acknowledgements: B.I.-M. and R.M.-A. is currently supported through the Project entitled “Platformă multidisciplinară de cercetare-dezvoltare medicală în regiunea N-E” (CENEMED, code: 127606), cofinanced through the Operational Program of Competitivity, Prioritary Axis: Research, Technological development, and Inovation (CDI).

**Disclosure of Interest:**

I. M. Balmus Grant / Research support from: Project entitled “Platformă multidisciplinară de cercetare-dezvoltare medicală în regiunea N-E”(acronym: CENEMED, mySMIS code: 127606), cofinanced through the Operational Program of Competitivity, Prioritary Axis: Research, Technological development, and Inovation (CDI)., M. -.-A. Robea Grant / Research support from: Project entitled “Platformă multidisciplinară de cercetare-dezvoltare medicală în regiunea N-E”(acronym: CENEMED, mySMIS code: 127606), cofinanced through the Operational Program of Competitivity, Prioritary Axis: Research, Technological development, and Inovation (CDI)., R. Lefter: None Declared, A. Ciobica: None Declared, L. Huiban: None Declared, C. Stanciu: None Declared, A. Trifan: None Declared

